# Energy Requirements for Growth in the Norfolk Terrier

**DOI:** 10.3390/ani11051380

**Published:** 2021-05-12

**Authors:** Sophie Bradley, Janet Alexander, Richard Haydock, Anne Marie Bakke, Phillip Watson

**Affiliations:** WALTHAM Petcare Science Institute, Waltham-on-the-Wolds, Melton Mowbray LE14 4RT, UK; janet.alexander@effem.com (J.A.); Richard.Haydock@effem.com (R.H.); anne.marie.bakke@effem.com (A.M.B.); Phillip.Watson@effem.com (P.W.)

**Keywords:** National Research Council, puppies, growth, small dog, BCS

## Abstract

**Simple Summary:**

Obesity and related diseases are common problems for dogs and inappropriate feeding during development is a contributor to life-long weight issues. Judging the right amount of food to give a growing puppy is challenging and providing a simple recommendation to owners is essential. However, differences in dog size, activity, and many other factors such as neutering can all have a role in impacting the actual energy requirements for growth. Yet, the current feeding guideline for growth (NRC 2006) does not accommodate these factors. Therefore, this study investigated how much a small breed (Norfolk Terrier puppies) requires to maintain growth and a healthy body condition score (BCS) through their first year of life. We found that they required significantly less than suggested by the NRC. Changes in the assessment of appropriate feeding during development are required and this study supports the need to revise the NRC (2006) equation for small breed dogs.

**Abstract:**

An appropriate energy intake for healthy growth can reduce the risk of obesity and co-morbidities, such as orthopaedic diseases. The 2006 National Research Council (NRC) universal equation calculates the energy requirement of growing dogs based on predicted adult body weight, but evidence suggests a revision may be required. This study investigates the energy requirements of seventeen Norfolk terrier puppies over their first year (10 to 52 weeks). Puppies were individually fed complete and balanced diets in amounts to maintain an optimal body condition score (BCS), recording intake daily and body weight and BCS weekly. To monitor health a veterinary examination, haematology and plasma biochemistry and serum measures of bone turnover were undertaken every 12 weeks. Skeletal development was assessed using dual-energy X-ray absorptiometry (26 and 52 weeks). Puppies were clinically healthy with normal skeletal development and healthy growth throughout. The energy intake to achieve this was significantly lower than that predicted by the NRC (2006) equation at all time points, with largest mean difference of 285 kJ/kg^0.75^ per day at 10 weeks. If fed according to the NRC 2006 equation, dogs would have been in positive energy balance, possibly leading to obesity. These data support a revision to the NRC (2006) equation.

## 1. Introduction

Obesity has been declared one of the greatest health challenges of the 21st century in our global pet population. A number of studies have revealed the scale of the problem in different regions of the world with the prevalence of overweight or obese dogs ranging from 20% to 50% [1,2,3,4,5,6]. This excessive bodyweight is linked to a myriad of associated conditions such as orthopaedic diseases [7,8,9,10], cardiorespiratory diseases [11,12], neoplasia [13,14] and diabetes mellitus [15,16,17]. As a result, the health-related quality of life and the expected lifespan are detrimentally affected [18,19].

Obesity is, however, preventable and can be controlled through managing energy intake, especially during early growth. Studies in humans have shown that atypical growth patterns during this critical window of life can predispose to obesity [20,21,22]. This predisposition to obesity has also been documented in cats where there are recommendations for energy intake to be tightly regulated after neutering to prevent unnecessary weight gain during early life. The authors demonstrate that if a healthy body weight and condition can be achieved during early life, then the risk of obesity in adult life is dramatically reduced [23]. Similarly, a dog study has shown that 83% of dogs that were overweight by early adulthood had crossed at least two centile lines when tracking their bodyweight through puppyhood using the WALTHAM Puppy Growth Charts [24]. Furthermore, studies have demonstrated that rapid weight gain during the first year of life is detrimental to skeletal development [25,26,27,28]. In a similar vein to cats, neutering also increases the risk of obesity in dogs in addition to sex, breed and owner characteristics/behaviours [2,3,4,29,30,31,32,33,34,35]. As such, healthy growth is essential during this critical timeframe and needs to be closely regulated.

Guidelines for the energy requirements during growth in puppies are provided by the National Research Council (NRC) in 2006 [36]. This was first suggested by Blanchard, Grandjean [37] and is in agreement with the equation suggested by Meyer and Zentek [38]. However, this is a single universal equation that does not take breed, sex, temperament, coat quality or neuter status into account. Previous studies demonstrated that the NRC [36] equation can result in an overestimation of their energy requirements, especially in younger puppies across a range of breeds [27,39,40]. These studies highlighted clear breed specific differences in energy requirements and a need for breed-specific feeding guides.

The objective of this study is to investigate the energy requirements for healthy growth in a small dog breed, the Norfolk terrier, from ten weeks to one year of age. This was achieved by feeding puppies to an optimum body condition score (BCS) and monitoring their growth trajectory, using WALTHAM™ Puppy Growth Charts. This was then compared to the NRC (2006) equation to determine whether this calculation is suitable to determine the energy requirements for healthy growth in this breed of dog.

## 2. Materials and Methods

### 2.1. Animals and Husbandry

This work was approved by the WALTHAM Animal Welfare and Ethical Review Body and conducted under the authority of the Animals (Scientific Procedures) Act 1986. A total of seventeen Norfolk Terriers from eight litters took part in the study. All puppies underwent a physical examination by a veterinary surgeon at the start and end of the study. Puppies were housed with their mother until weaning at 8 weeks of age, in litter groups until 10 weeks of age, and in pairs thereafter. In all cases, housing consisted of environmentally enriched kennels with constant access to an outdoor area. All puppies received socialisation and training sessions daily and access to large outdoor play areas. Free access to drinking water was provided at all times. Male puppies were neutered around 26 weeks and females remained entire throughout.

### 2.2. Diet Management

Puppies were offered a commercially available dry format diet (Royal Canin Yorkshire Terrier Junior; Mars Petcare) from multiple batches. Between the ages of 10 and 26 weeks, puppies were offered their daily ration in 3 × 30-minute meals equally spaced out between 8 am and 4 pm. From 27 to 52 weeks of age, the puppies received their daily intake across two meals across the same time frame. Diets underwent nutritional analysis (Eurofins, UK) to ensure compliance with the NRC and FEDIAF guidelines. In summary the average nutrient composition of the diets fed was as follows: Moisture = 20.6 g/4184 kJ; Protein = 72.4 g/4184 kJ; Fat = 48.8 g/4184 kJ; Ash = 17.3 g/4184 kJ; Crude fibre = 4.9 g/4184 kJ. Nutritional analysis results were used to calculate the average predicted metabolisable energy content of the diets (1701 kJ/100 g), which in turn was used to calculate the actual energy intake of the dogs.

### 2.3. Bodyweight Management

Food intake was recorded immediately following each meal as the mass of food offered minus the mass of food refused. Initial feeding allowances were determined by a veterinary surgeon through calculating each puppy’s resting energy requirements (RER) multiplied by three. After this point the amounts consumed were adjusted weekly, as needed (see below), with the aim of maintaining puppies at an optimum body condition score (BCS) throughout the study. Bodyweight was measured once per week, on the same weekday, using calibrated scales (Mettler-Toledo Ltd; Leicester; UK) throughout the trial. Although not fully validated in puppies, BCS was evaluated every week using a 9-point scale [41] by the same two assessors to maintain consistency. If the BCS of any puppy increased or decreased from an ideal BCS (score 4 or 5 out of 9), then the dietary amount was recalculated in order to achieve an ideal BCS. Dietary changes consisted of either a 5% increase if the BCS decreased from ideal (3 or less on 9-point BCS) or a 5% decrease if the BCS increased from ideal (6 or above on 9-point BCS). In addition, WALTHAM™ Puppy Growth Charts, a validated tool for healthy growth [42], were used to ensure a healthy growth trajectory of all of the puppies. Weekly bodyweights were plotted, and the growth trajectory monitored to stay within two centile lines. If the growth trajectory crossed two centile lines in either direction, then a 5% dietary increase or decrease was implemented.

### 2.4. Sample Collection and Analysis

At 3, 6, 9 and 12 months of age, fasted (>12 h) jugular blood samples were collected (2.2 mL total volume). Lithium–heparin-treated blood was centrifuged and the resulting plasma used for the determination of standard biochemistry parameters; total protein, albumin, inorganic phosphorus, alkaline phosphatase (ALP), alanine transaminase (ALT), aspartate aminotransferase (AST), calcium, cholesterol, urea, creatinine, triglycerides, sodium, potassium, chloride and glucose using an AU480 (Beckman Coulter (UK) Ltd; High Wycombe; UK) analyser. EDTA treated blood was collected for the measurement of standard haematology parameters using a 3-part differential automated haematology analyser (Mythic 18 Vet, Orphée, Geneva, Switzerland). Parameters measured were total leukocyte count, leucocyte counts as a number and percentage (lymphocytes, monocytes and granulocytes), total erythrocyte count, haemoglobin concentration, haematocrit, mean corpuscular volume, mean corpuscular haemoglobin, mean corpuscular haemoglobin concentration, erythrocyte distribution width, platelet count, and mean platelet volume. Markers of bone turnover were analysed in the serum: bone-specific alkaline phosphatase (BAP) and carboxy-terminal telopeptide cross-links (CTx) using MicroVue™ Quidel ELISA (TECO medical Group; Sissach; Switzerland) and CartiLaps^®^ ELISA (Immunodiagnostic Systems Ltd, Boldon; Tyne and Wear; UK.). Skeletal development was assessed at 26 and 52 weeks of age by means of Dual-energy X-ray absorptiometry (DXA; total body software package; Lunar Hologic QDR-1000 W; GE Healthcare; Chicago; USA). Prior to the scan, dogs were fasted at least 16 h and sedated with Torbugesic (0∙1 mg/kg; Pfizer Animal Health; Walton Oaks; Surrey; UK) and Dexmedetomidine (300μg/m2; Pfizer Animal Health; Walton Oaks; Surrey; UK) and reversed with Atipamezole (0∙1 mg/kg; Pfizer Animal Health, Walton Oaks; Surrey; UK).

### 2.5. Statistical Analysis

The predicted maintenance energy requirements were calculated, using the following NRC (2006) puppy energy requirement predictive equation (below), assuming that the adult bodyweight (kg) was that measured at 52 weeks of age.

MER(kcal / kg^0.75^) = 130 × 3.2(e^−0.87×(BW observed/BW52weeks)^ − 0.1) (1)

MER: maintenance energy requirements as recommended by the NRC 2006 guidelines, BW: Body weight.

Thereafter, kJ intake, NRC estimated requirements and the difference between them were modelled using linear mixed effects models. These models had fixed effects of age, sex and neuter status, and two-way interactions between neuter status and sex, and age. A random effect of dog nested in litter was also included to account for repeated measurements. Using these models mean values were estimated for each age, sex and neuter status combination. Likelihood ratio tests were used to test for the inclusion of the two-way interactions. Whilst their inclusion in the model was detected as statistically significant, visual inspection of the differences showed no biologically relevant difference. Means were, therefore, also estimated for each age averaged across levels of neuter status and sex to give an overall estimate. For the difference model (actual vs. NRC), all individual time point estimates were tested for a statistically significant difference from zero. All analyses were performed using R v3.5.1 [43] and the *lme4* [44], *multcomp* [45] and *lmerTest* [46] libraries. Single step multiple comparisons correction was performed to maintain a familywise error rate of 5%.

## 3. Results

**Figure 1:**
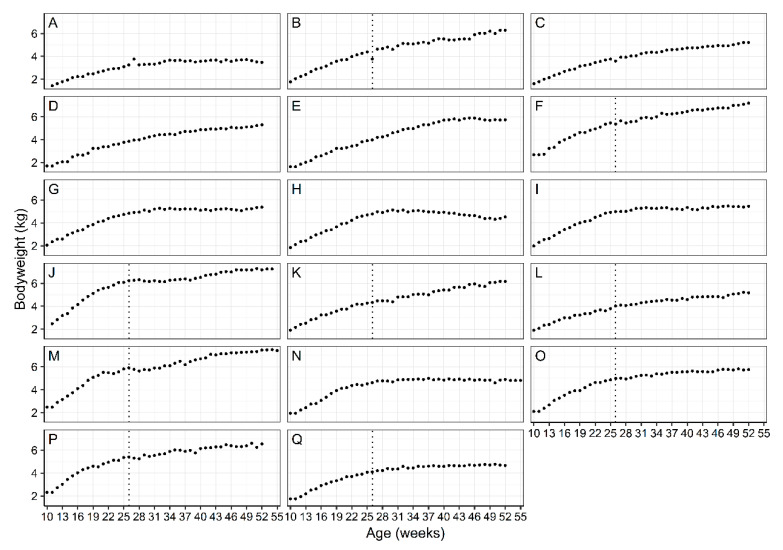
Body weight (kg) development with age for all Norfolk Terriers through the duration of the trial. (**A**–**Q**) designate the data for each of the 17 dogs. Dotted line depicts time of neutering which occurred in all male Norfolk Terriers around 26 weeks of age.

**Figure 2:**
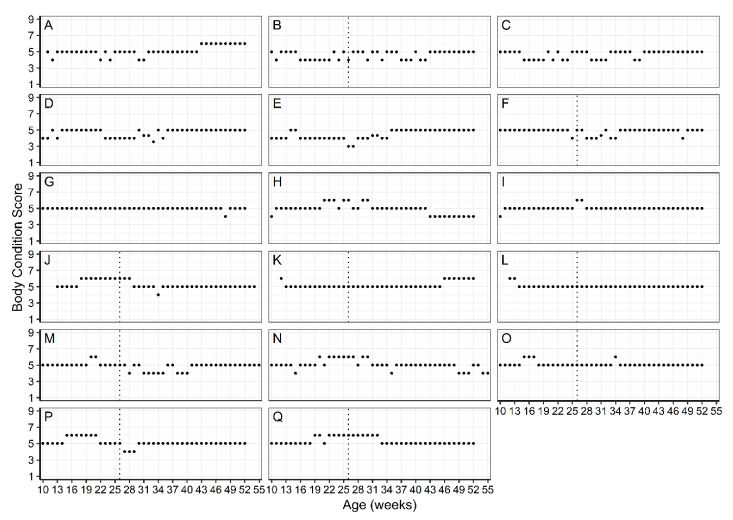
Weekly body condition scores of all Norfolk Terriers on the trial using the 9-point body composition scoring system. (**A**–**Q**) designate the data for each of the 17 dogs. Dotted line depicts the timing of neutering in all male Norfolk terriers.

All dogs remained healthy during the study, with no skeletal abnormalities observed, as judged by clinical examination and DXA. Haematological and biochemical parameters remained within normal ranges for all dogs (data not shown) and body weights increased with time for all dogs (Figure 1). Although some dogs deviated from an optimal BCS (4 or 5 out of 9) to a score of 3 or 6, this was corrected through dietary increases or decreases (Figure 2). At the end of the study, 1 male (K) and 1 female (A) were scored with a BCS of 6, while 15 dogs tracked at 4 or 5.

### 3.1. WALTHAM™ Puppy Growth Charts

**Figure 3:**
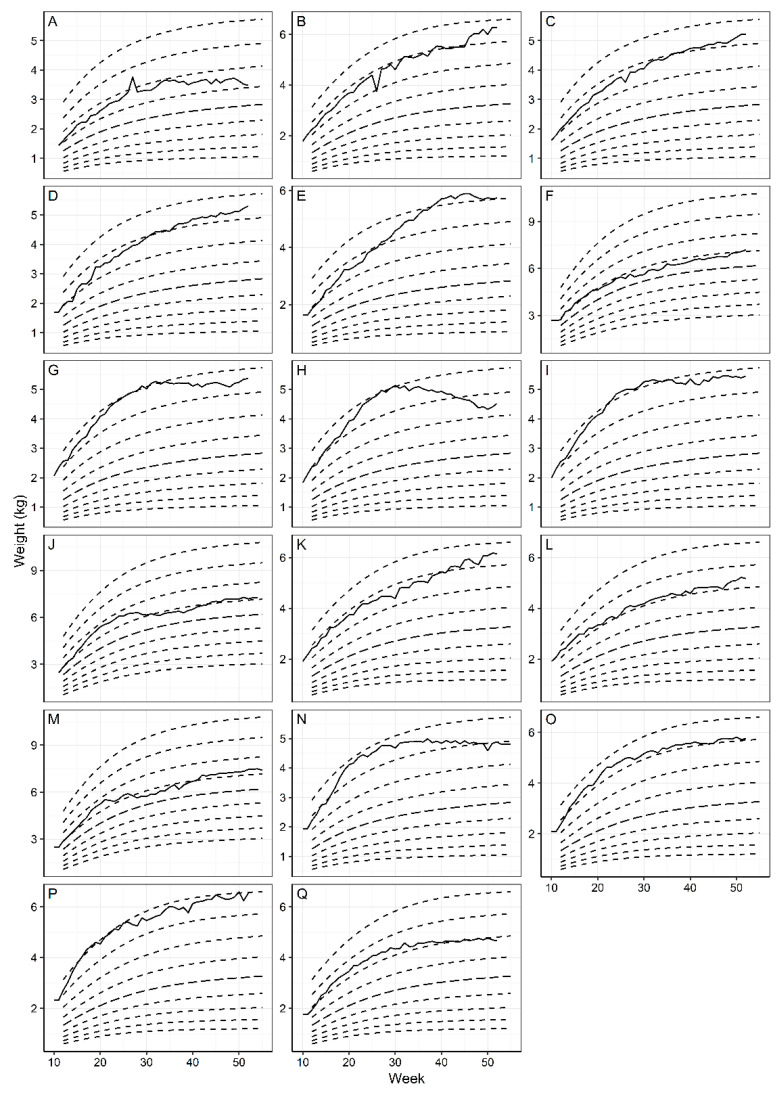
The growth trajectory for each dog was plotted throughout the study using WALTHAM Puppy Growth Charts. (**A**–**Q**) designate the data for each of the 17 dogs. Two different size charts were used based on the predicted adult bodyweight of each puppy. Charts (**A**–**E**), (**G**–**I**), (**K**–**L**) and (**N**–**Q**) were charts for <6.5 kg and charts (**F**,**J**,**M**) were charts for 6.5–9 kg. All but one dog (**E**) remained within two centile lines.

Growth was also tracked and recorded using the WALTHAM™ Puppy Growth Charts to ensure all dogs were growing in a healthy trajectory throughout the study (Figure 3). Only one dog, between 40 and 50 weeks (Figure 3E), crossed two centiles in an upward direction but this was managed through dietary intake reduction to bring the trajectory back to between two centiles. Although this is normal for some dogs, it can be indicative of rapid or compensatory growth, which could be relevant here as the BCS of this dog was the only BCS to drop to a 3 for a period of two weeks at week 26 and 27.

### 3.2. Energy Intake

**Figure 4:**
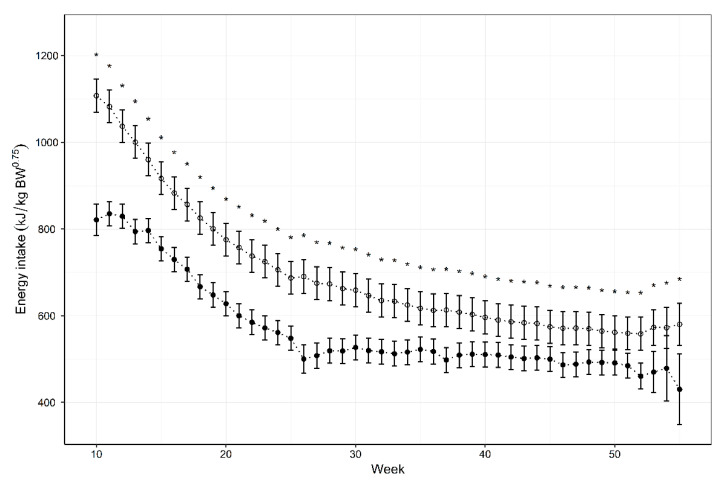
The energy intake of seventeen Norfolk Terriers (●) in comparison with the energy requirements predicted by the National Research Council (2006) equation (◦). Data are means, with 95 % confidence intervals represented by vertical bars. * Significant difference (*p* < 0.05).

The results indicate that the NRC (2006) equation overestimates the energy requirements throughout the first year (Figure 4). No impact of neuter status was observed on actual or predicted energy intake. When the data were split for sex difference (data not shown), the over estimation of energy intake was greater in males due to the NRC (2006) using the 52-week weight and adult males being heavier in comparison to females. Statistically significant differences (*p* < 0.05) were detected at all ages between the actual intakes and NRC estimates, when averaging across sex and neuter status. The maximum difference between the actual and predicted energy intake was a mean of 286 kJ/kg^0.75^ per day at 10 weeks of age. The difference reduced with age but the energy to maintain optimal body condition score always remained significantly less than the NRC predicted equation with the lowest difference being 71 kJ/kg^0.75^ per day at 50 weeks of age. (Table 1).

## 4. Discussion

The data presented here demonstrate that the NRC (2006) equation predicting energy requirements for growth consistently overestimates the energy requirements for Norfolk terrier puppies and is therefore not consistent with the energy required to maintain healthy development. Specifically, the data indicate that the NRC overestimates the daily energy needed for both male and female growing Norfolk terriers from 10 to 52 weeks of age. The energy intake needed to maintain a healthy growth trajectory and optimal body condition score was similar in both males and females, across the 52-week period. Neutering at 26 weeks did not significantly alter the energy intake needed for growth in male dogs. However, as all female dogs remained entire during this study, it cannot be determined whether neutering would significantly affect the energy requirements of female Norfolk terrier puppies. Sex differences and the interaction between neutering and risk of weight gain and obesity is currently unclear. Neutering can alter the level of circulating sex hormones, which have been shown to affect appetite regulation and metabolic rate, as well as altering levels of appetite-related hormones such as leptin and ghrelin [47,48,49,50,51,52,53]. However, most of the studies were conducted in cats. Canine specific studies are less numerous and somewhat contradictory with studies showing increased trends of food intake and body weight following neutering [54,55,56,57] and others showing no effect on food intake or bodyweight [58,59]. This could be explained through the difference in male and female sex hormones [60], which could result in differing food intake effects between sex post-neutering. Notwithstanding this, the incidence of obesity in adult dogs that are neutered is much higher than that of entire dogs [3,29,30,31,32,33,61]. However, many other factors are also related to canine obesity, such as age [2,31,33,61], breed [3,32], activity level [31,33,62] and owner characteristics and behaviour [62,63,64]. It is difficult to isolate or quantify the significance of each individual risk to obesity.

The energy intake data for the Norfolk terriers in this study is consistent with that previously reported for toy breed dogs living in a colony environment [23]. The estimated median MER was reported as 473 kJ/kg^0.75^ per day and the 52-week mean MER in the current study is 461 kJ/kg^0.75^ per day. Similarly, the average MER of pet dogs was demonstrated to be 519 ± 159 kJ/kg^0.75^ per day [65]. Importantly, the data presented here are consistent with studies that demonstrate collectively that the NRC recommendation overestimates the amount of energy required for healthy growth across multiple breeds [27,39,40,66,67]. Indeed, breed specific differences in energy requirements, especially between different sized breeds, have been documented, with Alexander et al. (2017) showing differences between the energy requirements of Yorkshire terriers, miniature schnauzers and Labrador retrievers. The energy intake per kg^0.75^ in Yorkshire terriers was significantly lower than both Labrador retrievers (until 29 weeks) and miniature schnauzers between (16 and 25 weeks). Furthermore, Dobenecker et al. (2013) showed a difference in energy requirements between foxhound-boxer-Ingelheim Labrador mixed breed puppies and beagle puppies up to 28 weeks of age. A large-scale study of client owned puppies reported that weaned puppies younger than 6 months had energy intakes that were approximately 80% of the NRC (2006) recommendation and in older puppies approximately 88% of this recommendation [39]. Collectively these studies suggest that breed differences in energy requirements should be considered when recommending feeding amounts during growth and the NRC (2006) universal equation is not suitable for this purpose.

Obesity is the fastest growing disease in both people and pets with an estimated 1.9 billion adult humans being overweight and a third of those obese [68]. Studies have drawn correlations between obese pet owners and obese pets [4,69,70] and with canine obesity being the number one health concern in dogs worldwide [33] interventions and management are now paramount. One critical intervention is to provide pet owners with accurate feeding guidelines to ensure adequate nutrition and energy intake. This is especially important to ensure healthy growth rates. Managing the energy requirements of dogs, especially during early growth, will also provide a healthy growth trajectory and body weight which will reduce the risk of obesity in adult life.

Throughout this study body condition scoring was used weekly to ensure the Norfolk terriers were being fed the correct energy requirements to maintain an optimal body condition. Although this method is widely used by veterinarians, researchers and some pet owners, due to the non-invasive nature and low cost, it is important to note that these scales are not validated for use in puppies and are a subjective tool. To reduce the subjectivity in this study, the assessment was conducted by the same two experienced assessors each week and a validated clinical tool for monitoring healthy growth was used in conjunction with BCS: the WALTHAM™ Puppy Growth Charts. These were developed using statistical modelling of the bodyweight and age data from 50,000 healthy dogs attending Banfield Pet Hospitals [42]. A recent study has shown that dogs who have crossed two centile lines on the growth charts, in either direction, were either over- or underweight by early adulthood, demonstrating the potential of this as a clinical tool for monitoring healthy growth in dogs [24]. Using these tools, this study provides additional evidence to demonstrate that feeding dogs to an optimal BCS results in healthy growth for the first year of life and also that the growth trajectory of the colony-held Norfolk terriers in this study is akin to client-owned dogs that the charts were modelled on.

A limitation of this current study is that we did not have a control group of puppies that were fed to the NRC (2006) equation for growth. The choice to not include a control group of this type was because previous evidence suggested that following the NRC recommendations would provide energy in excess of requirements to growing dogs possibly leading to overweight or obesity. Furthermore, this study shows that it is likely that if the dogs on this study had been fed to the NRC (2006) equation for growth, they would have been offered food in amounts providing excessive energy intake. This could have led to faster growth, likely at least partly due to increased fat deposition and resulted in possible harm to skeletal development and obesity [26,71,72,73]. The combined techniques of feeding to an optimal body condition score and using a WALTHAM™ Puppy Growth Chart to monitor weight development appeared to prevent or address any periods of rapid/slow growth before they become detrimental to the animal and thereby ensured healthy growth.

## 5. Conclusions

The data further support previous studies on energy requirements during growth in dogs and indicates the unsuitability of the NRC (2006) equation for the calculation of puppy energy requirements for Norfolk terriers. A re-evaluation of the NRC (2006) equation is required and this study supports the need for breed specific feeding guides for growth. Pet owners should be provided with the correct feeding guidelines for their pet to ensure adequate nutrition for a healthy life. The WALTHAM Puppy Growth Charts are an additional tool that could support pet owners and enable them to see their puppies’ growth trajectory in real time, again guiding pet owners towards providing healthy growth for their pet.

## Figures and Tables

**Table 1:** The monthly mean energy intake, NRC estimate and energy difference between the two measures, from seventeen Norfolk Terriers, are shown in the table below. Energy intakes are displayed as kJ/kg^0.75^ with 95% confidence intervals in brackets.

Age (Weeks)	Mean Energy Intake (kJ/kg^0.75^)	NRC Estimate (kJ/kg^0.75^)	Energy Difference (kJ/kg^0.75^)	*p*-Value
10	821.2 (784.7, 857.8)	1107.4 (1068.6, 1146.2)	−286.0 (−334.4, −237.6)	<0.001
14	796.7 (768.7, 824.7)	960.1 (922.5, 997.8)	−163.3 (−204.9, −121.6)	<0.001
18	667.0 (639.0, 695.1)	825.7 (788.1, 863.4)	−158.6 (−200.2, −116.9)	<0.001
22	585.1 (557.1, 613.1)	737.8 (700.1, 775.4)	−152.5 (−194.2, −110.9)	<0.001
26	500.3 (467.3, 533.4)	690.4 (651.7, 729.0)	−190.0 (−235.9, −144.2)	<0.001
30	527.1 (498.4, 555.7)	658.9 (620.8, 697.0)	−131.9 (−174.4, −89.5)	<0.001
34	516.2 (487.6, 544.8)	624.8 (586.7, 662.9)	−108.7 (−151.1, −66.3)	<0.001
38	509.1 (480.5, 537.7)	608.5 (570.3, 646.6)	−99.6 (−142.0, −57.2)	<0.001
42	504.8 (476.2, 533.4)	586.2 (548.1, 624.3)	−81.7 (−124.1, −39.2)	<0.001
46	486.7 (458.1, 515.4)	571.1 (533.0, 609.3)	−84.7 (−127.1, −42.2)	<0.001
50	491.4 (462.8, 520.1)	561.8 (523.7, 599.9)	−70.5 (−113, −28.1)	<0.001
52	460.6 (430.8, 490.5)	558.3 (520.0, 596.5)	−97.8 (−141.2, −54.5)	<0.001

## Data Availability

The data presented in this study are available on request from the corresponding author.
